# Host Long Noncoding RNAs as Key Players in Mycobacteria–Host Interactions

**DOI:** 10.3390/microorganisms12122656

**Published:** 2024-12-21

**Authors:** Stephen K. Kotey, Xuejuan Tan, Audrey L. Kinser, Lin Liu, Yong Cheng

**Affiliations:** 1 Department of Biochemistry and Molecular Biology, Oklahoma State University, Stillwater, OK 74078, USA; stephen.k.kotey@okstate.edu (S.K.K.); xtan9@okstate.edu (X.T.); audrey.dagnell@okstate.edu (A.L.K.); 2 Oklahoma Center for Respiratory and Infectious Diseases, Oklahoma State University, Stillwater, OK 74078, USA; lin.liu@okstate.edu; 3 Department of Physiological Sciences, Oklahoma State University, Stillwater, OK 74078, USA

**Keywords:** long noncoding RNAs, lncRNAs, mycobacterial infections, *Mycobacterium tuberculosis*, *Mycobacterium bovis* BCG, non-tuberculous mycobacteria, macrophages, host cells

## Abstract

Mycobacterial infections, caused by various species within the Mycobacterium genus, remain one of the main challenges to global health across the world. Understanding the complex interplay between the host and mycobacterial pathogens is essential for developing effective diagnostic and therapeutic strategies. Host long noncoding RNAs (lncRNAs) have emerged as key regulators in cellular response to bacterial infections within host cells. This review provides an overview of the intricate relationship between mycobacterial infections and host lncRNAs in the context of *Mycobacterium tuberculosis* and non-tuberculous mycobacterium (NTM) infections. Accumulation of evidence indicates that host lncRNAs play a critical role in regulating cellular response to mycobacterial infection within host cells, such as macrophages, the primary host cells for mycobacterial intracellular survival. The expression of specific host lncRNAs has been implicated in the pathogenesis of mycobacterial infections, providing potential targets for the development of novel host-directed therapies and biomarkers for TB diagnosis. In summary, this review aims to highlight the current state of knowledge regarding the involvement of host lncRNAs in mycobacterial infections. It also emphasizes their potential application as novel diagnostic biomarkers and therapeutic targets.

## 1. Introduction

Mycobacterial infections are caused by bacterial pathogens belonging to the genus *Mycobacterium*, which includes various species capable of infecting humans and animals. The two main categories of mycobacteria relevant to infections in humans are the *Mycobacterium tuberculosis* complex (MTBC) and non-tuberculous mycobacteria (NTM). The MTBC includes *Mycobacterium tuberculosis* (*M. tuberculosis*), *Mycobacterium bovis*, and *Mycobacterium africanum* [1]. NTM consists of a diverse group of environmental mycobacterial species, such as the *Mycobacterium avium* complex (MAC) and *Mycobacterium abscessus* complex (MABC) [2,3,4,5]. These NTM species constitute opportunistic mycobacterial pathogens capable of causing pulmonary infections. The MABC and MAC are accountable for 80–85% of lung infectious diseases caused by NTM infection worldwide [6]. On the contrary, the MTBC is obligate pathogens with no known biological reservoir aside from humans or animals [7]. Mycobacterial species including the MTBC and NTMs can invade many other organs aside from the lungs causing extrapulmonary infections like osteomyelitis, meningitis, and thoracic lymph nodes infections [7].

*M. tuberculosis* is the causative agent of tuberculosis (TB), a contagious disease in humans. About one-third of the world’s population are infected with *M. tuberculosis*. Among those infected with *M. tuberculosis*, about 90–95% of the population live with a status called latent TB, which refers to the long-term persistence of *M. tuberculosis* within the human host. The remaining 5–10% of those infected with *M. tuberculosis* develop active TB disease during their lifetime, leading to about 1.4 million deaths annually, including HIV-positive patients [8]. TB is primarily a pulmonary disease and transmitted through aerosol droplets containing *M. tuberculosis*. Once inhaled, these aerosol droplets reach and are deposited in lung alveoli, and *M. tuberculosis* is then taken up by alveolar macrophages. *M. tuberculosis* has evolved the ability to survive and replicate within macrophages in the lung [9]. Therefore, the interaction between *M. tuberculosis* and alveolar macrophages determines the consequence of *M. tuberculosis* infection (clearance, active, or latent TB) in the lung. Both host and mycobacterial factors, such as the macrophage activation state, the host genetics, and mycobacterial virulent molecules that evade the host’s defense mechanism in macrophages, are determinant in the outcome of *M. tuberculosis* infection. After the initial infection, the host begins to build up an acquired immunity against *M. tuberculosis*. The activated monocytes/macrophages, neutrophils, primed T and B lymphocytes, and other immune cells migrate to the infection site in the lung and help in the elimination of the pathogen. These types of immune cells further contribute to the formation of granuloma, a hallmark of the immune response to *M. tuberculosis* infection in the lung of TB patients. If the granuloma is broken down over time, exponential proliferation of *M. tuberculosis* occurs in macrophages, followed by cell lysis, and the release of intracellular bacteria and infection in new macrophages. The bacteria are then able to spread into the airways and be transmitted to the next host through the air as aerosol droplets. Additionally, *M. tuberculosis* also can spread throughout the body via circulatory and lymphatic systems, causing extrapulmonary TB such as meningeal TB [10,11,12].

As we described above, the majority of *M. tuberculosis* infections exist as a latent TB in humans, meaning the pathogen persists in the lung of the infected person for a long time and even a lifetime. Under certain conditions, such as compromised immune system or aging, latent TB can convert to active TB [13,14]. To control a global TB pandemic, the World Health Organization (WHO) launched the Stop TB Initiative in 2000, to bring together partners from various countries to accelerate efforts in controlling and eliminating TB as a global health threat [15]. Therefore, it becomes necessary to understand the mechanism of *M. tuberculosis*–host interactions, and identify host and mycobacterial factors that regulate the progression of *M. tuberculosis* infection in the host.

Different from *M. tuberculosis*, NTM species widely exist in the environment and mainly infect individuals with compromised immune systems or pre-existing lung conditions, such as the elderly and patients with chronic obstructive pulmonary disease (COPD) or cystic fibrosis (CF) [2,3,4,5]. NTM can infect different tissues and organs in humans, particularly leading to respiratory and skin diseases. The clinical presentation varies widely, and diagnosis involves molecular biology tools or isolating the specific NTM species through bacterial cultures. NTM species are becoming an emerging group of bacterial pathogens in CF patients with lung diseases [16,17]. However, the treatment for NTM infections can be challenging due to the natural resistance of NTM species to many antibiotics and the need for prolonged therapy [2,3]. To better control NTM infections in CF, it is necessary to understand the mechanisms by which the patients with CF are more susceptible to NTM lung infection.

Long noncoding RNAs (lncRNAs) are a class of RNA molecules that are longer than 200 nucleotides and lack protein-coding potential. Unlike messenger RNAs (mRNAs), which serve as templates for protein synthesis, lncRNAs play diverse regulatory roles in various cellular processes, such as regulating gene expression and epigenetic modification, binding and regulating protein function, and interacting with microRNAs [18]. Increasing evidence indicates that host lncRNAs are engaged in the cellular response to microbial infections in the host [19,20,21,22]. However, there are only limited studies understanding the role of host lncRNAs in cellular response to mycobacterial infection within host cells. In this review, we will summarize the current progress in the research on host lncRNAs and mycobacterial infections, mainly focusing on *M. tuberculosis* and NTM.

## 2. Host LncRNAs

Until the discovery of the first eukaryotic lncRNA, *H19*, most genetics research was mainly focused on elucidating and characterizing the protein-coding regions of the genome [23]. Further light was shed on the importance of lncRNAs when the lncRNA *XIST* (X-inactive specific transcript) was identified to regulate cellular pathways in eukaryotic cells, attracting researchers to the field of lncRNA studies. In the past three decades, a growing body of research increased our understanding of the biological functions of the lncRNA genes in eukaryotic cells. Early studies on the genomic DNA structure and sequence resulted in the conclusion that the largest proportion of the genome was made up of sequences lacking any biological function since they did not code for proteins. Thus, these sequences were given the term selfish or junk DNA for the span of about 20 years [24]. The Human Genome Project (HGP) and the FANTOM and RIKEN consortia transcriptome analysis in humans and mice further revealed that some transcripts (ncRNAs: noncoding RNAs) within human cells were independent of the protein-coding genes in the genome [23,25,26,27,28]. By employing DNA arrays and cDNA sequence analysis, these ncRNA transcripts were mapped to the mouse and human reference genomes. Using Cap Analysis Gene Expression (CAGE) sequencing, it was discovered that two-thirds of the mouse genome were ncRNA genes, and ncRNAs were transcribed from either sense or antisense DNA strands [29].

LncRNAs are one type of ncRNA that have been identified in all species including animals, plants, fungi, bacteria, and viruses [23]. They have been an expansive repertoire of ncRNAs engaged in the numerous biological processes, and disease pathogenesis and progression [30,31]. The development of RNA sequencing and artificial intelligence prediction technologies has led to an increase in the identification of lncRNAs in humans and experimental animal models [32]. lncRNAs generally have more than 200 nucleotides and are expressed at a low level. Within mammalian cells, lncRNAs can be found in the nucleus where they regulate gene expression, or in the cytosol to regulate certain cellular pathways by binding to the proteins or miRNAs [32,33]. In comparison to mRNA, lncRNAs show fewer similarities across species, and lack a significant number of introns [34]. But similarly, lncRNAs are transcribed by polymerase II, and modified by 5′-cap and 3′-polyA tail [35].

LncRNAs have been classified based on their location in the genome from which they are synthesized, their collective functions, and/or their subcellular location [36]. On the matter of their original genomic locations, they are categorized under five classes: sense, antisense, bidirectional, intronic, and intergenic lncRNAs [36,37]. Sense lncRNAs are those synthesized from a protein-coding gene in the same direction of mRNA transcription whereas the antisense type follows the opposite direction of mRNA transcription; bidirectional lncRNA originates from a close proximity (within 1 kb) to the promoter of an associated protein-coding gene and is synthesized in the opposite direction of the mRNA; intronic lncRNA as the name suggests, is transcribed within the intron of an associated protein-coding gene whereas intergenic lncRNA arises from the region between two protein-coding genes (i.e., the intergenic region) [37].

## 3. Host lncRNAs and Mycobacterial Infections

**Figure 1:**
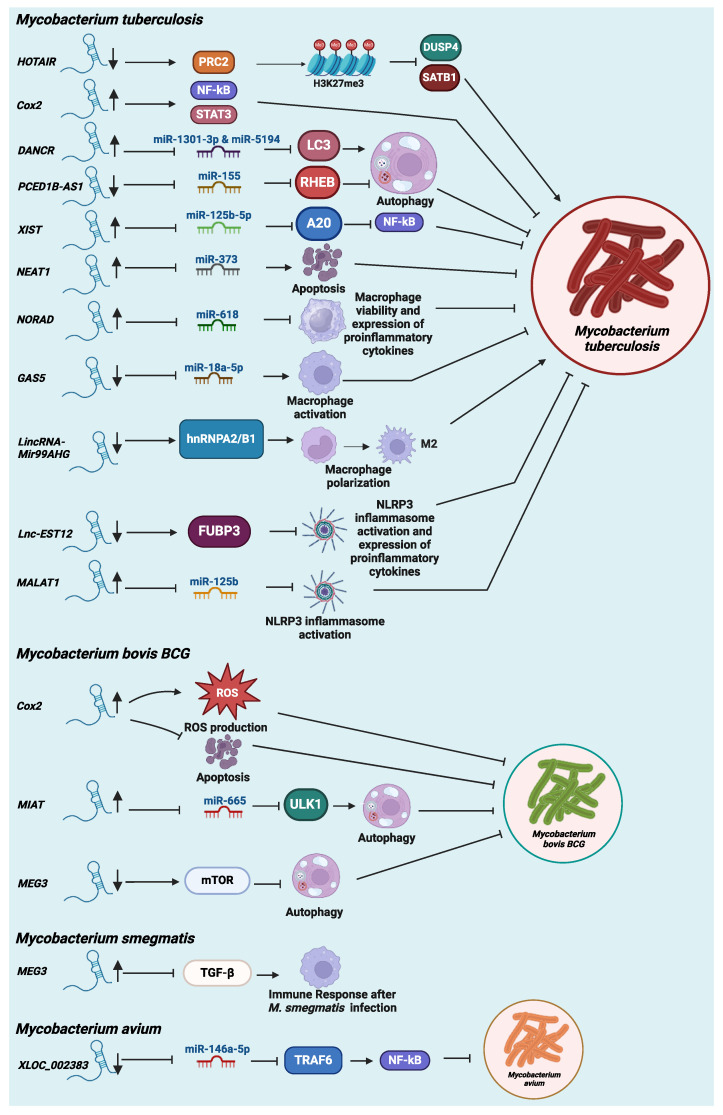
Host lncRNAs in mycobacterial infections. PRC2: Polycomb repressive complex 2; DUSP4: Dual-specificity protein phosphatase 4; SATB1: Special AT-rich sequence-binding protein-1; NF-κβ: Nuclear factor kappa B; STAT3: Signal transducer and activator of transcription 3; LC3: Microtubule-associated protein 1A/1B-light chain 3 (MAP1LC3B); RHEB: Ras homolog enriched in brain; A20: TNF alpha induced protein 3 (TNFAIP3); hnRNPA2/B1: Heterogeneous nuclear ribonucleoproteins A2/B1; FUBP3: Far upstream element-binding protein 3; ULK1: Unc-51-like autophagy-activating kinases 1; mTOR: Mammalian target of rapamycin; TGF-β: Transforming growth factor beta; TRAF6: TNF receptor-associated factor 6.

Various studies have shown that host small noncoding regulatory RNAs like microRNAs are critical in host response to mycobacterial infection in both cell culture and mouse models [38]. In addition to this class of well-studied ncRNAs, the engagement of host lncRNAs in the mycobacteria–host interactions is gradually coming to the light of understanding. Thus, it is of no surprise that lncRNAs have also been revealed to play critical roles in host response to mycobacteria infections, suggesting a potential application of host lncRNAs as a novel host-directed therapy for mycobacterial infections. In the following section, we summarized host lncRNAs that have been identified to play critical roles in host responses to *M. tuberculosis* or NTM infections in host cells, especially in macrophages (Figure 1).

### 3.1. Host lncRNAs and M. tuberculosis Infection 

**LncRNA *HOTAIR*** (HOX Transcript Antisense RNA) is transcribed from the *HOXC* gene locus in chromosome 12 in human and mouse genomes. It has been found that lncRNA *HOTAIR* is involved in cell pattern formation and differentiation by interacting with the PRC2 (Polycomb Repressive Complex 2) components and subsequently facilitating their recruitment to chromatin [39,40,41]. PRC2 is crucial in sustaining the repressed condition of the *HOX* genes and acts as an important epigenetic chromatin modifier that transfers three methyl groups to lysine 27 of histone H3, i.e., H3K27me3, thus silencing the gene [39,42]. lncRNA *HOTAIR* was the first lncRNA identified as a trans-regulatory element for gene repression in the HOXD locus at chromosome 2 [39]. It has been shown that in conjugation with histone modifications, lncRNA *HOTAIR* regulates the expression of host genes that control the survival of virulent *M. tuberculosis* within macrophages [41]. In this study, it was found that lncRNA *HOTAIR* increases H3K27me3 modification on the transcription start sites (TSSs) of the DUSP4 (Dual specific MAP kinase phosphatase 4) and SATB1 (Specific AT-rich sequence-binding protein 1) genes, and represses their expression within macrophages. DUSP4 and SATB1 are critical for the survival of *M. tuberculosis* within macrophages, and decrease the expression and release of neutrophil-recruiting cytokines CXCL1, CXCL2, and CXCL3. Interestingly, the expression of lncRNA *HOTAIR* is downregulated by virulent *M. tuberculosis* H37Rv compared to avirulent *M. tuberculosis* H37Ra within macrophages [41]. It suggests that *M. tuberculosis* H37Rv has evolved a survival strategy by interfering with the expression of lncRNA *HOTAIR* within macrophages. However, the regulatory mechanism remains unclear.

***LincRNA-Cox2*** (Cyclooxygenase-2) promotes the polarization of macrophages to the pro-inflammatory M1 phenotype known to be well primed for *M. tuberculosis* killing. It was recently found that lincRNA*-Cox2* is important for NF-κβ and Stat3 activation to limit the intracellular *M. tuberculosis* survival in human macrophages [43]. The NF-κβ and Stat3 are two critical transcriptional factors that mediate cellular response to *M. tuberculosis* infection in macrophages. The transcriptional factor NF-κβ is mainly involved in the initiation of pro-inflammatory response that yields antimicrobial responses, and other biological processes like cell proliferation, metastasis, DNA damage response, and programmed cell death [44,45]. The mechanism by which *lincRNA-Cox2* regulates NF-κB activation in macrophages during *M. tuberculosis* infection remains unclear. However, a recent study has suggested that *lincRNA-Cox2* may bind to the NF-κB p65 subunit, promoting its nuclear translocation. This likely facilitates the formation of a transcriptional activation complex, ultimately controlling the expression of immune-related genes, such as the inflammasome sensor NLRP3 and the adaptor protein ASC in BV2 cells, mouse BMMs, and primary microglial cells [46]. STAT3, on the other hand, helps control inflammation but lessens the antimicrobial responses if overly expressed [47]. Known as the “anti-inflammatory STAT”, STAT3 regulates macrophage polarization through the JAK/STAT signaling pathway after its activation via phosphorylation [47,48,49]. In addition to its effect on controlling *M. tuberculosis* infection within macrophages, *lincRNA-Cox2* knockdown was found to increase apoptosis in RAW264.7 cells that were infected with *M. bovis* BCG, an attenuated strain of *Mycobacterium bovis* and the only licensed vaccine for human TB [50], by regulating cellular pathways associated with apoptosis, inducing ROS production and activating endoplasmic reticulum (ER) stress signals [51]. Interestingly, it was recently shown that *lincRNA-Cox2* functions as a cis or trans immune-regulatory factor for gene expression. As a cis factor, *lincRNA-Cox2* acts as an enhancer RNA to regulate the expression of the *Ptgs2* gene, which encodes cyclooxygenase-2 (Cox2), a key enzyme in the prostaglandin biosynthesis pathway [52]. Knockdown of Cox2 downregulates autophagy in mouse macrophages [53]. Given the critical role of autophagy in the killing of *M. tuberculosis*, it would be valuable to explore whether *lincRNA-Cox2* also promotes autophagy in response to *M. tuberculosis* infection in macrophages.

**LncRNA-*MIAT*** (Myocardial infarction-associated transcript). With studies showing *MIAT*’s involvement in the activation of inflammatory response in various diseases, lncRNA-*MIAT* has attracted the attention of the researchers in the field of infectious diseases [54,55]. This lncRNA has been found to facilitate autophagosome formation and intracellular mycobacteria clearance in macrophages. Infection of THP-1 cells with *M. bovis* BCG led to an upregulated expression of lncRNA-*MIAT* which was subsequently identified to facilitate apoptosis and macrophage autophagy. Through the lncRNA’s role as a competitive endogenous miRNA sponge to miR-665, it enhances the expression of ULK1 (Unc-51 like autophagy-activating kinase 1) and restricts the intracellular growth and proliferation of *M. bovis* BCG within macrophages [56,57]. ULK1 is a well-known mediator of cell death and plays a critical role in autophagy, apoptosis, and necrosis [58,59]. It would be interesting to investigate if/how lncRNA-*MIAT* regulates autophagy, apoptosis, and necrosis in macrophages via ULK1 in response to *M. tuberculosis* infection.

**LncRNA-*DANCR*** (Differentiation antagonizing non-protein coding RNA) is located at the locus 4q12.5 in the human genome and was initially found to promote the expression of IL-6 and TNF-α, thus inducing pro-inflammatory response within immune cells [60]. In the context of *M. tuberculosis* infection, the expression of lncRNA-*DANCR* is highly elevated in TB patients compared to healthy individuals. Similarly, the expression of lncRNA-*DANCR* was induced in human THP-1-derived macrophages that were infected with the nonvirulent *M. tuberculosis* strain, H37Ra. It was further found that lncRNA-*DANCR* upregulates autophagy and restrains intracellular mycobacterial survival within macrophages by sponging the autophagy inhibitors miR-1301-3p and miR-5194, which target host mRNAs encoding autophagy-associated proteins [56,57].

**LncRNA *PCED1B-AS1*** (PC-esterase domain containing 1B antisense RNA 1). Similar to lncRNA-*MIAT* and lncRNA-*DANCR*, lncRNA *PCED1B-AS1* also regulates apoptosis and autophagy within macrophages during *M. tuberculosis* infection. However, the expression of lncRNA *PCED1B-AS1* is downregulated in monocytes obtained from active TB patients correlating with inhibited apoptosis and induced autophagy in human macrophages [61]. Moreover, it was found that lncRNA *PCED1B-AS1* regulates apoptosis and autophagy by acting as a miRNA sponge for miR-155. Its interception of miR-155 liberates the miRNA’s effect on apoptosis inhibition and autophagy enhancement through the proteins FOXO3 and Rheb, respectively [62,63]. The downregulation of lncRNA *PCED1B-AS1* expression during *M. tuberculosis* infection seems advantageous for the proliferation of the intracellular *M. tuberculosis* since apoptosis inhibition serves to protect the bacteria from immune attack; however, the interplay also seems beneficial to the host cells through the facilitation of autophagosome formation that serves to restrict intracellular *M. tuberculosis* proliferation. Therefore, this study suggests a complexity of host lncRNA actions in the host–pathogen interactions during a mycobacterial infection.

**LncRNA *XIST*** (X-inactive-specific transcript) is a lncRNA that has been indicated to play an important role in *M. tuberculosis* survival within mouse and human macrophages by interacting with miR-125b-5p [64]. In this study, it was found that the expression of lncRNA *XIST* in mouse RAW 264.7 cells and human monocyte-derived macrophages (hMDMs) was induced by *M. tuberculosis* infection via a mycobacterial EsxA-dependent pathway. As a result, miR-125b-5p, one of the targets of lncRNA *XIST*, fails to repress the A20 protein (an NF-κB repressor) and to activate downstream NF-κb activation in macrophages. Interestingly, the expression of lncRNA *XIST* is also induced in the lung, spleen, and lymph nodes of mice that were infected with *M. tuberculosis* for 12 weeks. Knockdown of lncRNA *XIST* significantly attenuated *M. tuberculosis* survival within mouse RAW 264.7 cells and hMDMs in cell culture. In contrast, overexpression of lncRNA *XIST* facilitated *M.* tuberculosis survival within mouse and human macrophages. Negative pressure therapy in tuberculosis has been known to be an effective treatment in alleviating the TB disease. It involves the administration of a uniformly distributed vacuum across a wound surface to eliminate liquid discharge from wounds, decrease interstitial swellings, seal wound edges, and enhance delivery of blood to the wound. By exerting mechanical strain on the wound, this therapy activates cells responsible for regulating the microenvironment around the wound site [65,66,67]. In the same study, Luo et al. showed that the lncRNA *XIST* expression within mouse RAW 264.7 cells and hMDMs was significantly downregulated after negative pressure treatment in cell culture. Consequently, miR-125b-5p-mediated NF-κb activation is rescued, ultimately promoting pro-inflammatory M1 macrophage polarization [64].

**LncRNA-*NEAT1*** (Nuclear-enriched abundant transcript 1) is a lncRNA that has the potential to be a biomarker for *M. tuberculosis* infection and TB treatment outcome since it has been found that the expression level of lncRNA-*NEAT1* increased during the course of *M. tuberculosis* infection and had a significant impact on the survival of *M. tuberculosis* within THP-1-derived macrophages [68]. While it remains unclear how lncRNA-*NEAT1* regulates antimycobacterial response in macrophages, it has been demonstrated that there was a positive correlation between the expression of lncRNA-*NEAT1* and the level of IL-6 within macrophages, suggesting a pro-inflammatory mechanism against *M. tuberculosis* [68]. Moreover, a similar correlation was observed in samples from patients suffering from spinal tuberculosis [69], suggesting IL-6 plays a key mediatory role in the lncRNA-*NEAT1*-mediated immune response in tuberculosis. More recently, it was shown that lncRNA-*NEAT1* inhibits cellular apoptosis and facilitates macrophage proliferation via regulating miR-373 during *M. tuberculosis* infection in cell culture [70]. Finally, the expression level of lncRNA-*NEAT1* correlates with clinical characteristics in patients suffering from spinal tuberculosis, such as segments of ulcerations, cysts adjacent to the spinal column, anti-TB regimen, drug resistance, the level of C-reactive protein, and the rate of erythrocyte sedimentation (ERS) [68,69]. It suggests a potential application of lncRNA-*NEAT1* as a biomarker for the progression of tuberculosis and outcome of TB treatment.

**LncRNA-*NORAD*** (noncoding RNA activated by DNA damage) is another host lncRNA explored in human pulmonary tuberculosis (PTB). An increased level of lncRNA-*NORAD* was detected in the serum of PTB patients compared to healthy individuals, correlating with an increased level of the pro-inflammatory cytokines IL-1β, TNF-α, and IL-6 [71]. Similarly, the expression of lncRNA-*NORAD* was highly upregulated by *M. tuberculosis* infection in mouse RAW 264.7 cells and human THP-1-derived macrophages. It was further identified that lncRNA-*NORAD* improves cell viability and pro-inflammatory response by acting as a miRNA sponge for miR-618, an inhibitor of macrophage viability and activation, in RAW 264.7 cells and THP-1-derived macrophages during *M. tuberculosis* infection [71]. Therefore, the results above indicate a potential role of lncRNA-*NORAD* in host defense against *M. tuberculosis* infection in macrophages.

**LncRNA-*GAS5*** (Growth arrest-special transcript 5) is another host lncRNA associated with intracellular *M. tuberculosis* growth by inhibiting host cell viability and downregulating the production of pro-inflammatory cytokines, such as TNF-α, IL-1β, and IL-6, in macrophages [72]. It is interesting to note that the expression level of lncRNA-*GAS5* is downregulated in the serum of latent and active TB patients and human THP-1 cells after infection with *M. tuberculosis*. As proposed in this study, lncRNA-*GAS5* likely interferes with host cell viability and cellular response to *M. tuberculosis* infection by serving as a miRNA sponge for miR-18a-5p [72].

**LincRNA-*MIR99AHG*** is a host lncRNA that is downregulated during *M. tuberculosis* infection and engaged in *M. tuberculosis* survival within mouse and human macrophages [73]. In this work, Gcanga et al. discovered that the expression of lincRNA-*MIR99AHG* was regulated by the macrophage polarization state, showing upregulation in M2 polarized macrophages and downregulation in M1 polarized cells. Moreover, its expression was downregulated by *M. tuberculosis* infection through the NF-κB pathway since NF-κB inhibitor, Bay11–7082, restored the expression levels of lincRNA-*MIR99AHG* in mouse macrophages that were infected with *M. tuberculosis* in cell culture. LincRNA-*MiR99AHG* knockdown led to a defect in the growth of intracellular *M. tuberculosis* along with a reduction in the production of pro-inflammatory cytokines and necrosis whilst facilitating early apoptosis in mouse macrophages in cell culture. Consistently, lincRNA-*MiR99AHG* knockdown also reduced *M. tuberculosis* survival and pro-inflammatory responses in human monocyte-derived macrophages in cell culture and in the lung of mice. It was further discovered that lincRNA-*MIR99AHG* was translocated to the nucleus where it bound with heterogeneous nuclear ribonucleoprotein, hnRNPA2/B1, which has been identified to regulate macrophage polarization. Interestingly, hnRNPA2/B1 also interacts with lincRNA-*COX2*, another important host lncRNA in macrophages in response to *M. tuberculosis* infection [43,74]. Therefore, it suggests that there is some degree of crosstalk between host lncRNAs, such as lincRNA-*MIR99AHG and* lincRNA-*COX2*, in macrophages during *M. tuberculosis* infection.

***Lnc-EST12*** (lncRNA Early Secreted Parget with a molecular weight of 12 kDa) is primarily expressed in immune-associated organs like the liver, lung, and spleen, and its expression is downregulated by *M. tuberculosis*-secreted protein EST12 in mouse RAW 264.7 macrophages in a time-dependent manner [75]. It has been found that *M. tuberculosis* EST12 represses host *lnc-EST12* expression via the JAK2-STAT5a signaling pathway by which phospho-STAT5a is translocated to the nucleus and binds to the promoter region of the *lnc-EST12* gene, subsequently inhibits its expression. By binding to the KH3 and KH4 domains of the transcription factor FUBP3 (Far Upstream Element-binding protein 3), *lnc-EST12* guides FUBP3 binding to the promoter sites of the pro-inflammatory cytokines such as IL-1β and IL-6, resulting in an attenuated expression of pro-inflammatory cytokines and antimycobacterial cellular response in macrophages. Additionally, the *lnc-EST12-*FUBP3 complex also represses EST12-induced assembly of NLRP3 inflammasome and Gasdemin D-mediated pyroptosis, two important cellular pathways in responses to *M. tuberculosis* infection in macrophages [75,76].

**lncRNA-*MALAT1*** (Metastasis-associated lung adenocarcinoma transcript 1), also known as *NEAT2* (Nuclear-Enriched Abundant Transcript 2), regulates various cellular processes such as oxidative stress, cellular pyroptosis, and inflammation in host cells [77,78]. This host lncRNA has been studied earlier as a novel biomarker for TB diagnosis [79]. More recently, it was recognized that lncRNA-*MALAT1* aids in NLRP3 inflammasome activation and cellular pyroptosis by acting as a sponge for host miR-125b that downregulates the production of TLR4 in macrophages in response to *M. tuberculosis* infection [78].

### 3.2. Host lncRNAs and NTM Infections

Once thought to be harmless, NTM infection-related global mortality has steadily been rising [80]. In particular, they cause severe infections in the individuals with compromised immune systems or pre-existing pulmonary conditions, such as the elderly and the patients with COPD or CF [2,3,4,5,81]. Similar to TB, very few studies have been published in understanding the engagement of host lncRNAs in host–NTM interactions (Figure 1).

**LncRNA-*MEG3*** (Maternally expressed gene 3) is a lncRNA that has been extensively studied for its engagement in the progression of various human diseases [82]. It was recently identified that the expression of lncRNA-*MEG3* was induced within THP-1-derived macrophages during *Mycobacterium smegmatis* infection. It correlates with a decreased expression of TGF-β, a pivotal regulator of the immune response [83]. It has been found previously that lncRNA-*MEG3* regulated the expression of the TGF-β gene by formation of an RNA–DNA complex which recruited distal regulatory elements and thus caused a transcriptional repression of the TGF-β gene in breast cancer cells [84]. Different from *M. smegmatis* infection, Pawar et al. observed that the expression of lncRNA-*MEG3* was downregulated by *M. bovis* BCG infection in THP-1-derived macrophages in cell culture. Their study further showed that lncRNA-*MEG3* is associated with mTOR and PI3K-AKT signaling pathways. Knockdown of lncRNA-*MEG3* significantly increases autophagy and intracellular *M. bovis BCG* killing within human macrophages [85]. Therefore, the data suggest that mycobacterial pathogens have likely evolved the unique regulatory strategy to manipulate the expression of host lncRNA-*MEG3* which contributes to the outcome of mycobacterial infections in the host.

**LncRNA *XLOC_002383*** was revealed to possess anti-mycobacterial activity against *M. avium* in THP-1 cells by endogenously competing for tumor necrosis factor receptor-associated factor (TRAF6) with miR-146a-5p, resulting in the secretion of pro-inflammatory cytokines [86]. TRAF6 is a critical component in host TLR-mediated signaling pathways and regulates the activation of the downstream molecules such as mitogen-activated protein kinase (MAPK) and NF-κB, which contribute to the production of pro-inflammatory cytokines [87]. Yan et al. recently showed that miR-146a-5p was able to suppress inflammation and improve healing of airway epithelial cells by targeting TRAF6 [88]. As a result, by acting as a sponge for miR-146a-5p, LncRNA *XLOC_002383* increases the production of TRAF6 and thus elevates the expression of pro-inflammatory cytokines such as TNF-α, IL-1β and IL-6 in macrophages in response to mycobacterial infection. As expected, the activation of the lncRNA *XLOC_002383*/miR-146a-5p/TRAF6 pathway reduces intracellular *M. avium* survival in THP-1-derived macrophages. Interestingly, the expression of lncRNA *XLOC_002383* declined over time post *M. avium* infection in THP-1-derived macrophages [86]. It suggests that *M. avium* has evolved a survival mechanism which inhibits the expression of antimycobacterial host lncRNAs.

**Other lncRNAs.** Investigation of lncRNA profiles in bovine macrophages in response to *Mycobacterium avian* subspecies *parartuberculosis* (MAP) infection revealed several novel host lncRNAs, including *XLOC_000633*, *XLOC_030080*, and *XLOC_029370*. Their expression levels were highly upregulated in macrophages in response to mycobacterial infection in cell culture. Interestingly, these lncRNAs are co-expressed with neighboring protein-encoding genes, suggesting their potential regulatory roles in the expression of neighboring genes and immune response. The regulatory mechanisms they are involved in remain unknown [89,90].

### 3.3. Host lncRNAs in Intercellular Communication During Mycobacterial Infection

Extracellular vesicles (EVs) are nanoscale membrane-bound particles released by various cell types into the extracellular environment. They play a crucial role in intercellular communication by transferring proteins, lipids, DNA, and RNA between cells, thereby regulating numerous physiological and pathological processes in recipient cells [91]. EVs released by mammalian cells can be classified into different types, including exosomes and microvesicles, based on their biogenesis and size. These vesicles play a significant role in modulating host response to bacterial infections. Exosomes are initially formed as intraluminal vesicles (ILVs) in multivesicular bodies (MVBs) via the inward budding of late endosomes. ILVs are then released to the extracellular environment as exosomes after the fusion of MVBs with cytoplasmic membrane of parental cells. Microvesicle biogenesis involves the outward budding and fission of the plasma membrane, leading to the release of microvesicles from the parental cell [92,93]. While it is less studied, accumulating evidence indicates that mammalian lncRNAs can be packaged into EVs and subsequently delivered to recipient cells, where they can influence various biological processes, including gene expression, cellular differentiation, and immune responses [94,95]. For example, Li et al., identified 1022 lncRNAs via exLR-seq (extracellular vesicle long RNA sequencing) in the EVs that were isolated from the plasma of a cohort of 260 healthy individuals [96]. Under certain disease conditions, EV-carried lncRNAs may regulate tissue pathology. A recent study indicates that hypoxic cardiomyocyte-released EVs mediate the function of fibroblasts through its cargo, *lncRNA-NEAT1*, and play an important role in cardiac fibrosis [97]. In response to *M. tuberculosis* or NTM infection, macrophages or neutrophils release EVs that contain molecules from both host and mycobacteria, including proteins, DNA, RNA, and lipids [98,99,100,101,102,103,104]. These host-cell-derived EVs regulate the function of recipient cells, such as macrophages, neutrophils, and T cells, and affect the host immunity in response to mycobacterial infection in vitro and in vivo [17,103,105,106,107,108,109,110,111]. For example, EVs from *M. tuberculosis*-infected macrophages carry mycobacterial RNAs and activate the cytosolic RNA sensor RIG-I-dependent LC3-associated phagocytosis (LAP) pathway, leading to *M. tuberculosis* killing in recipient macrophages [103,104]. As mentioned earlier, host lncRNAs play a central role in the host’s response to mycobacterial infection in host cells. Given the involvement of EV-carried lncRNAs in the progression of human diseases like cardiac fibrosis [97], it suggests that host-cell-released EVs likely also carry host lncRNAs that contribute to cell–cell communication in the context of mycobacterial infection. Therefore, it would be important to explore the role of EV-carried host lncRNAs in the interaction between the host and mycobacterial pathogens.

### 3.4. Host lncRNAs as Biomarkers for TB Diagnosis

Identifying host lncRNAs as potential biomarkers for TB diagnosis has become a prominent area of various studies that intend to improve the accuracy and early detection of the disease [112,113]. While this field is still in its nascent stage, a number of studies have shown that the expression patterns of particular host lncRNAs have been linked with the persistence, evolution, treatment, diagnosis, and prognosis of TB [113].

As described above in this review, TB infection regulates the expression of a number of host lncRNAs in immune cells, especially in macrophages. Chen et al. employed a microarray analysis and identified a large number of host lncRNAs (163 upregulated and 348 downregulated) altered in the plasma of TB patients, including *NR_038221*, *NR_003142*, *ENST00000570366*, and *ENST00000422183* [112]. The pathway analysis indicated that these host lncRNAs were associated with T-cell regulation in line with other works that have demonstrated the impact of host lncRNAs on the function of CD4^+^ and CD8^+^ lymphocytes in TB patients [114,115]. These differentially expressed lncRNAs would be potential diagnostic markers for TB.

The change in lncRNA abundance is also seen in the studies that have sought to emphasize the exploitation of lncRNAs in distinguishing active TB cases from healthy individuals. For example, the level of lncRNA-*NEAT1* was found to be highly upregulated in peripheral blood mononuclear cells (PBMCs) and granulomatous tissue from patients with spinal tuberculosis (STB), as well as in THP-1 cells after *M. tuberculosis* infection in cell culture [68]. Additionally, the expression level of lncRNA-*NEAT1* was significantly associated with clinical characteristics in patients with STB, including paraspinal abscesses, segments of the lesions and anti-TB treatment, the level of IL-6 and C-reactive protein, and erythrocyte sedimentation rate. Further evidence indicated that the expression level of lncRNA-*NEAT1_1*, one of the lncRNA-*NEAT1* isoforms, predicted good prognosis of STB and might become a prognostic biomarker for STB [69]. More recently, using Receiver Operating Characteristic (ROC) curves, the lncRNAs *LINC00152* and *LARS2-AS1* proved to be legitimate biomarkers in not only distinguishing latent TB cases from healthy ones but also active TB cases from latent TB, albeit moderately [116]. In a separate study, the neutrophil lncRNA *ZNF100-6:2* also showed good diagnostic potential after examining it through ROC analyses. It was found that the level of lncRNA *ZNF100-6:2* increased over time in active TB patients receiving anti-TB treatment. It suggests that lncRNA *ZNF100-6:2* might be used as novel markers for TB diagnosis and outcome of anti-TB treatment [117].

The application of host lncRNAs as TB biomarkers has the advantage of identifying TB disease even in the absence of microbiological evidence for *M. tuberculosis* [113]. According to the WHO report in 2017, only 57% of PTB cases reported to the WHO in 2016 were bacteriologically confirmed, implying that about half of the cases are not clinically diagnosed or are false negatives for smear microscopy [118]. Moreover, clinically symptomatic cases of PTB likely lack confirmatory results of *M. tuberculosis* infection from smear microscopy analysis, mycobacterial culture and/or PCR amplification tests. It is urgent to develop a complementary diagnostic technique to aid in the detection of *M. tuberculosis* infection. To develop a predictive model to facilitate clinical diagnoses of PTB, Hu et al. designed a web-based nomogram based on the differentially expressed host lncRNAs in the patients with PTB and electronic health records (EHRs) [119]. Their PTB diagnosis model showed a greater level of capability identifying PTB cases amongst presumed patients with negative microbiological evidence for *M. tuberculosis*.

In light of the advantages, there are some challenges that interfere with the use of host lncRNAs as diagnostic tools in TB and push forth the need for further exploration in this field. This includes lncRNA’s proneness to degradation due to the instability of RNAs as a result of the free 2′–hydroxyl group on the pentose ring that makes it susceptible to hydrolysis as well as the ubiquitous nature of RNA degradative enzymes. Additionally, despite the great specificity of lncRNAs to distinguish TB cases from healthy individuals in the studies, many studies have not been performed to seek the specificity of lncRNAs in distinguishing one disease from the other [20,120]. This is important since most of the lncRNAs mentioned in this review also play significant roles in other diseases or in response to the infections caused by other microbial pathogens. For example, in addition to its regulation in TB pathogenesis, lncRNA *HOTAIR* has been implicated in colorectal and cervical cancers [121,122]. LincRNA*-Cox2* regulates inflammatory responses in the diseases like hepatocellular carcinoma and sepsis [123,124]. LncRNA*-NEAT1* has been found to have a mediatory role in Parkinson’s disease, sepsis, and cancers [125,126,127]. This brings up a challenge in identifying disease-specific lncRNAs for the diagnosis of TB cases and presents a research area to push the boundaries of this field.

## 4. Conclusions and Future Perspective

Host lncRNAs play pivotal roles in the cellular response to microbial infections within host cells while the engagement of most host lncRNAs in host–pathogen interactions remains unknown. Further studies of host lncRNAs involved in mycobacterial infections might be the key in understanding the ‘missing links’ that come into play in the host–pathogen interactions in the context of mycobacterial infections. The differentially expression profile of host lncRNAs in response to mycobacterial infections along with their association with mycobacterial survival in the host and cellular processes such as autophagy, apoptosis, proliferation, etc., marks them as ideal targets for the development of novel diagnosis tools and host-directed therapy for mycobacterial infections in patients with TB or NTM infections. Future research on host lncRNAs should focus on validating potential lncRNA candidates and exploring their use alongside other TB diagnostic tools. It should also aim to develop user-friendly, cost-effective diagnostic assays and continue investigating the functional roles of host lncRNAs in TB pathogenesis, particularly in animal models, to provide deeper insights into novel therapeutic targets. Finally, the application of genome editing techniques, such as CRISPR/Cas systems, along with artificial intelligence, holds great potential for uncovering the role of lncRNAs in the host response to mycobacterial infection.
